# Supplementary material for: Multi-gene phylogeny and divergence estimations for Evaniidae (Hymenoptera)
Source: PeerJ. 2019 Apr 4;7:e6689. doi: 10.7717/peerj.6689 (PMC6451838; doi:10.7717/peerj.6689)
Supplement: Table S1 — Exemplars used from Deans, Gillespie & Yoder (2006) are listed with the reference from that paper (Deans ID) beside the internal voucher number (Extract ID). Included genes for each taxon are marked with an x. Gene codes: 28S for 28S rDNA; AM2 for alpha-mannosidase II; CAD1 and CAD2 for carbamoyl-phosphate sythetase-asparate transcarbamoylase-dihydroorotase (CAD) (for amplicon regions for each segment, see Fig. 1); RPS23 for Ribosomal Protein S23; COI for cytochrome oxidase I; and 16S for 16S rDNA. [file peerj-07-6689-s003.docx]

**Supplementary Material**:

**Table S1**. Taxonomic and Genetic Sampling. Exemplars used from Deans et al. ([2006](#_ENREF_3)) are listed with the reference from that paper (Deans ID) beside the internal voucher number (Extract ID). Included genes for each taxon are marked with an x. Gene codes: 28S for 28S rDNA; AM2 for alpha-mannosidase II; CAD1 and CAD2 for carbamoyl-phosphate sythetase-asparate transcarbamoylase-dihydroorotase (CAD) (for amplicon regions for each segment, see Figure 1); RPS23 for Ribosomal Protein S23; COI for cytochrome oxidase I; and 16S for 16S rDNA.

|  |  | **Taxon** | **Extract ID** | | **Deans ID** | | **28S** | | **AM2** | | **CAD 1** | | **CAD 2** | | **RPS 23** | | **CO1** | | **16S** | | **No. Genes** | |  |
| --- | --- | --- | --- | --- | --- | --- | --- | --- | --- | --- | --- | --- | --- | --- | --- | --- | --- | --- | --- | --- | --- | --- | --- |
| **Outgroup** | | |  | |  | |  | |  | |  | |  | |  | |  | |  | |  | |  |
|  | ***Gasteruption* Latreille** | |  | |  | |  | |  | |  | |  | |  | |  | |  | |  | |  |
|  |  | *Gasteruption* sp.300 | 300 | |  | | x | |  | | x | | x | | x | | x | | x | | 6 | |  |
|  |  | *Gasteruption* sp.244 | 244 | |  | | x | |  | | x | |  | | x | | x | |  | | 4 | |  |
|  | ***Pristaulacus* Kieffer** | |  | |  | |  | |  | |  | |  | |  | |  | |  | |  | |  |
|  |  | *Pristaulacus strangaliae* | 176 | |  | |  | | x | |  | |  | | x | | x | |  | | 3 | |  |
|  |  | *Pristaulacus fasciatus* | 299 | |  | |  | |  | |  | |  | |  | | x | |  | | 1 | |  |
|  |  | *Pristaulacus* sp.021 | 306 | | 021 | | x | |  | |  | |  | | x | | x | | x | | 4 | |  |
| **Evaniidae** | | |  | |  | |  | |  | |  | |  | |  | |  | |  | |  | |  |
|  | ***Acanthinevania* Bradley** | |  | |  | |  | |  | |  | |  | |  | |  | |  | |  | |  |
|  |  | *Acanthinevania* sp.240 | 240 | |  | | x | | x | | x | | x | | x | | x | |  | | 6 | |  |
|  |  | *Acanthinevania* sp.242 | 242 | |  | | x | | x | | x | | x | | x | |  | |  | | 5 | |  |
|  |  | *Acanthinevania princeps* (Westwood) | 246 | |  | | x | |  | | x | | x | | x | | x | |  | | 5 | |  |
|  |  | *Acanthinevania* sp.001 | 271 | | 001 | | x | | x | | x | | x | | x | | x | | x | | 7 | |  |
|  |  | *Acanthinevania* sp.033 | 289 | | 033 | | x | | x | | x | | x | | x | | x | | x | | 7 | |  |
|  |  | *Acanthinevania* sp.049 | 292 | | 049 | | x | | x | | x | | x | | x | | x | | x | | 7 | |  |
|  | ***Alobevania* Deans and Kawada** | |  | |  | |  | |  | |  | |  | |  | |  | |  | |  | |  |
|  |  | *Alobevania gattiae* | 200 | | 039 | | x | | x | | x | | x | |  | | x | | x | | 6 | |  |
|  | ***Brachygaster* Leach** | |  | |  | |  | |  | |  | |  | |  | |  | |  | |  | |  |
|  |  | *Brachygaster minutus* (Olivier) | 273 | | 030 | | x | |  | | x | | x | | x | | x | | x | | 6 | |  |
|  |  | *Brachygaster minutus* (Olivier) | 512 | |  | |  | |  | | x | | x | |  | | x | |  | | 3 | |  |
|  |  | *Brachygaster* sp.037 | 286 | |  | | x | |  | | x | |  | | x | |  | | x | | 4 | |  |
|  |  | *Brachygaster* sp.050 | 290 | |  | | x | |  | |  | |  | | x | | x | | x | | 4 | |  |
|  | ***Decevania* Huben, 2003** | |  | |  | |  | |  | |  | |  | |  | |  | |  | |  | |  |
|  |  | *Decevania* sp.502 | 502 | |  | |  | |  | | x | | x | |  | |  | |  | | 2 | |  |
|  |  | *Decevania* sp.513 | 513 | |  | |  | | x | |  | |  | |  | | x | |  | | 2 | |  |
|  |  | *Decevania* sp.004 | 274 | | 004 | | x | | x | |  | | x | | x | | x | | x | | 6 | |  |
|  |  | *Decevania* sp.005 | 301 | | 005 | | x | |  | |  | |  | | x | |  | | x | | 3 | |  |
|  |  | *Decevania* sp.063 | 296 | | 063 | | x | | x | | x | | x | | x | | x | | x | | 7 | |  |
|  | ***Evania* Fabricius** | |  | |  | |  | |  | |  | |  | |  | |  | |  | |  | |  |
|  |  | *Evania* sp.175 | 175 | |  | | x | |  | |  | |  | | x | | x | |  | | 3 | |  |
|  |  | *Evania albofacialis* Cameron | 275 | | 020 | | x | |  | | x | | x | | x | | x | | x | | 6 | |  |
|  |  | *Evania appendigaster* (Linnaeus) | 207 | | 046 | | x | |  | | x | | x | | x | | x | | x | | 6 | |  |
|  |  | *Evania* sp.496 | 496 | |  | | x | |  | |  | | x | | x | | x | |  | | 4 | |  |
|  |  | *Evania* sp.002 | 189 | | 002 | | x | |  | | x | | x | |  | | x | | x | | 5 | |  |
|  | ***Evaniella* Bradley** | |  | |  | |  | |  | |  | |  | |  | |  | |  | |  | |  |
|  |  | *Evaniella* sp.230 | 230 | |  | | x | | x | | x | | x | | x | | x | |  | | 6 | |  |
|  |  | *Evaniella* sp.234 | 234 | |  | | x | | x | |  | | x | | x | | x | |  | | 5 | |  |
|  |  | *Evaniella* sp.237 | 237 | |  | |  | | x | | x | |  | |  | | x | |  | | 3 | |  |
|  |  | *Evaniella* sp.485 | 485 | |  | |  | | x | | x | | x | | x | |  | |  | | 4 | |  |
|  |  | *Evaniella* sp.486 | 486 | |  | |  | | x | | x | |  | | x | |  | |  | | 3 | |  |
|  |  | *Evaniella* sp.493 | 493 | |  | |  | |  | | x | |  | | x | | x | |  | | 3 | |  |
|  |  | *Evaniella semaeoda* Bradley | 220 | | 058 | | x | |  | |  | |  | | x | | x | | x | | 4 | |  |
|  |  | *Evaniella* sp.019 | 192 | | 019 | | x | | x | | x | |  | | x | | x | |  | | 5 | |  |
|  |  | *Evaniella* sp.025 | 307 | | 025 | | x | |  | | x | |  | | x | | x | | x | | 5 | |  |
|  |  | *Evaniella* sp.045 | 206 | | 045 | | x | | x | | x | |  | | x | |  | | x | | 5 | |  |
|  | ***Evaniscus* Sze´pligeti** | |  | |  | |  | |  | |  | |  | |  | |  | |  | |  | |  |
|  |  | *Evaniscus marginatus* (Cameron) | 213 | | 052 | | x | |  | |  | |  | | x | |  | | x | | 3 | |  |
|  |  | *Evaniscus rufithorax* Enderlein | 287 | |  | | x | |  | |  | | x | | x | | x | | x | | 5 | |  |
|  | ***Hyptia* Illiger** | |  | |  | |  | |  | |  | |  | |  | |  | |  | |  | |  |
|  |  | *Hyptia* sp.232 | 232 | |  | |  | | x | | x | | x | | x | | x | |  | | 5 | |  |
|  |  | *Hyptia* sp.487 | 487 | |  | |  | | x | |  | |  | |  | | x | |  | | 2 | |  |
|  |  | *Hyptia* sp.501 | 501 | |  | |  | | x | | x | |  | |  | | x | |  | | 3 | |  |
|  |  | *Hyptia* sp.511 | 511 | |  | |  | | x | | x | |  | |  | | x | |  | | 3 | |  |
|  |  | *Hyptia amazonica* (Schletterer) | 235 | |  | |  | |  | | x | | x | |  | | x | |  | | 3 | |  |
|  |  | *Hyptia floridana* Ashmead | 291 | | 009 | | x | | x | | x | | x | |  | | x | | x | | 6 | |  |
|  |  | *Hyptia* sp.007 | 302 | | 007 | | x | |  | | x | |  | | x | | x | | x | | 5 | |  |
|  |  | *Hyptia* sp.008 | 303 | | 008 | | x | |  | |  | |  | | x | | x | | x | | 4 | |  |
|  | ***Micrevania* Benoit** | |  | |  | |  | |  | |  | |  | |  | |  | |  | |  | |  |
|  |  | *Micrevania difficilis* Benoit | 283 | | 006 | | x | |  | | x | | x | |  | | x | | x | | 5 | |  |
|  |  | *Micrevania* sp.061 | 288 | | 061 | | x | | x | |  | | x | |  | | x | | x | | 5 | |  |
|  |  | *Micrevania* sp.066 | 298 | | 066 | | x | |  | |  | | x | |  | | x | | x | | 4 | |  |
|  |  | *Micrevania* sp.026 | 308 | | 026 | | x | |  | |  | | x | |  | | x | | x | | 4 | |  |
|  | ***Papatuka* Deans** | |  | |  | |  | |  | |  | |  | |  | |  | |  | |  | |  |
|  |  | *Papatuka capensis* (Schletterer) | 227 | | 065 | | x | |  | | x | | x | | x | | x | | x | | 6 | |  |
|  | ***Parevania* Kieffer** | |  | |  | |  | |  | |  | |  | |  | |  | |  | |  | |  |
|  |  | *Parevania* sp.172 | 172 | |  | | x | | x | | x | |  | | x | |  | |  | | 4 | |  |
|  |  | *Parevania* sp.174 | 174 | |  | | x | | x | |  | |  | | x | | x | |  | | 4 | |  |
|  |  | *Parevania* sp.041 | 295 | | 041 | | x | | x | | x | | x | | x | | x | | x | | 7 | |  |
|  |  | *Parevania* sp.057 | 219 | | 057 | | x | | x | | x | | x | | x | |  | | x | | 6 | |  |
|  |  | *Parevania* sp.064 | 276 | | 064 | | x | |  | | x | | x | | x | | x | | x | | 6 | |  |
|  | ***Prosevania* Kieffer** | |  | |  | |  | |  | |  | |  | |  | |  | |  | |  | |  |
|  |  | *Prosevania fuscipes* (Illiger) | 224 | | 062 | | x | |  | |  | | x | |  | | x | | x | | 4 | |  |
|  |  | *Prosevania* sp.497 | 497 | |  | | x | | x | | x | | x | |  | | x | |  | | 5 | |  |
|  |  | *Prosevania* sp.498 | | 498 | |  | |  | |  | | x | |  | |  | | x | |  | | 2 | |
|  |  | *Prosevania* sp.508 | | 508 | |  | |  | |  | |  | |  | |  | | x | |  | | 1 | |
|  |  | *Prosevania* sp.027 | | 309 | | 027 | | x | | x | |  | | x | |  | | x | | x | | 5 | |
|  |  | *Prosevania* sp.034 | | 277 | | 034 | | x | |  | |  | |  | |  | | x | | x | | 3 | |
|  |  | *Prosevania* sp.036 | | 284 | | 036 | | x | |  | | x | | x | |  | | x | | x | | 5 | |
|  |  | *Prosevania* sp.044 | | 205 | | 044 | | x | | x | | x | | x | | x | | x | | x | | 7 | |
|  | ***Rothevania* Huben** | | |  | |  | |  | |  | |  | |  | |  | |  | |  | |  | |
|  |  | *Rothevania valdivianus* (Philippi) | | 239 | | 048 | | x | | x | | x | | x | |  | | x | | x | | 6 | |
|  | ***Semaeomyia* Bradley** | | |  | |  | |  | |  | |  | |  | |  | |  | |  | |  | |
|  |  | *Semaeomyia* sp.489 | | 489 | |  | |  | |  | | x | | x | | x | | x | |  | | 4 | |
|  |  | *Semaeomyia* sp.509 | | 509 | |  | | x | |  | |  | | x | | x | | x | |  | | 4 | |
|  |  | *Semaeomyia* sp.510 | | 510 | |  | | x | |  | |  | | x | | x | | x | |  | | 4 | |
|  |  | *Semaeomyia leucomelas* (Kieffer) | | 305 | | 016 | | x | |  | |  | | x | | x | | x | | x | | 5 | |
|  |  | *Semaeomyia* sp.012 | | 197 | | 012 | | x | |  | | x | | x | |  | | x | | x | | 5 | |
|  |  | *Semaeomyia* sp.051 | | 279 | | 051 | | x | |  | |  | | x | | x | | x | | x | | 5 | |
|  |  | *Semaeomyia* sp.059 | | 293 | | 059 | | x | | x | |  | | x | | x | | x | | x | | 6 | |
|  | ***Szepligetella* Bradley** | | |  | |  | |  | |  | |  | |  | |  | |  | |  | |  | |
|  |  | *Szepligetella* sp.170 | | 170 | |  | |  | |  | | x | |  | | x | | x | |  | | 3 | |
|  |  | *Szepligetella* sp.231 | | 231 | |  | | x | |  | | x | | x | | x | | x | |  | | 5 | |
|  |  | *Szepligetella* sp.233 | | 233 | |  | | x | | x | | x | | x | | x | | x | |  | | 6 | |
|  |  | *Szepligetella* sp.236 | | 236 | |  | | x | | x | | x | | x | |  | | x | |  | | 5 | |
|  |  | *Szepligetella* sp.238 | | 238 | |  | | x | | x | | x | | x | | x | | x | |  | | 6 | |
|  |  | *Szepligetella* sp.241 | | 241 | |  | |  | |  | | x | | x | | x | | x | |  | | 4 | |
|  |  | *Szepligetella* sp.243 | | 243 | |  | | x | | x | | x | |  | | x | | x | |  | | 5 | |
|  |  | *Szepligetella* sp.247 | | 247 | |  | |  | |  | | x | |  | | x | | x | |  | | 3 | |
|  |  | *Szepligetella* sp.248 | | 248 | |  | | x | |  | | x | |  | | x | | x | |  | | 4 | |
|  |  | *Szepligetella sericea* | | 297 | |  | |  | |  | |  | | x | | x | | x | |  | | 3 | |
|  |  | *Szepligetella* sp.047 | | 208 | | 047 | | x | | x | | x | |  | | x | | x | | x | | 6 | |
|  |  | *Szepligetella* sp.055 | | 280 | | 055 | |  | | x | | x | |  | | x | | x | | x | | 5 | |
|  |  | *Szepligetella* sp.056 | | 294 | | 056 | | x | | x | | x | | x | | x | | x | | x | | 7 | |
|  |  | *Szepligetella* sp.285 | | 285 | |  | |  | | x | | x | | x | | x | | x | |  | | 5 | |
|  | ***Trissevania* Kieffer** | | |  | |  | |  | |  | |  | |  | |  | |  | |  | |  | |
|  |  | *Trissevania anemotis* Kieffer | | 282 | | 038 | | x | | x | |  | | x | | x | | x | | x | | 6 | |
|  |  | *Trissevania* sp.507 | | 507 | |  | |  | |  | |  | | x | |  | |  | |  | | 1 | |
|  | ***Zeuxevania* Kieffer** | | |  | |  | |  | |  | |  | |  | |  | |  | |  | |  | |
|  |  | *Zeuxevania* sp.499 | | 499 | |  | |  | |  | | x | | x | |  | |  | |  | | 2 | |
|  |  | *Zeuxevania* sp.500 | | 500 | |  | |  | |  | | x | | x | |  | | x | |  | | 3 | |
|  |  | *Zeuxevania* sp.503 | | 503 | |  | |  | |  | |  | | x | |  | |  | |  | | 1 | |
|  |  | *Zeuxevania* sp.505 | | 505 | |  | | x | |  | | x | | x | | x | | x | |  | | 5 | |
|  |  | *Zeuxevania* sp.015 | | 191 | | 015 | | x | |  | | x | |  | |  | | x | | x | | 4 | |
|  |  | *Zeuxevania splendidula* (Costa) | | 312 | | 031 | | x | |  | | x | | x | | x | | x | | x | | 6 | |
|  |  | Percent Amplified | |  | |  | | 71 | | 44 | | 66 | | 64 | | 67 | | 86 | | 50 | |  | |
|  |  | Percent Parsimony Informative Sites | |  | | 40 | | 44 | | 49 | | 53 | | 35 | | 60 | | 40 | |  | |  | |
