# Supplementary material for: Multi-gene phylogeny and divergence estimations for Evaniidae (Hymenoptera)
Source: PeerJ. 2019 Apr 4;7:e6689. doi: 10.7717/peerj.6689 (PMC6451838; doi:10.7717/peerj.6689)
Supplement: Table S2 — For protein-coding genes, the Primer Name is based off the amino acid position in the Apis mellifera CDS for that gene. An Alternate Primer Name is given if a different name was used in a previously published study or in-house. [file peerj-07-6689-s004.docx]

**Table S1**. **Primers and PCR protocols used in this study**. For protein-coding genes, the Primer Name is based off the amino acid position in the *Apis mellifera* CDS for that gene. An Alternate Primer Name is given if a different name was used in a previously published study or in-house.

| **Gene** | **Primer Name** | **Alternate Primer Name** | **Primer Sequence (5' to 3')** | **PCR Cycling Conditions** | **Source** | **Comments** |
| --- | --- | --- | --- | --- | --- | --- |
|  |  |  |  |  |  |  |
| 28S | 28SD2D3F |  | AGAGAGAGTTCAAGAGTACGTG | 94°C⁄ 3min; [94°C⁄ 30s; 52°C⁄ 30s; 72°C⁄ 70s]x35; 72°C⁄ 7min | Dowton & Austin (2001) |  |
|  | 28SD2D3R |  | TTGGTCCGTGTTTCAAGACGGG |  | Dowton & Austin (2001) |  |
|  | 28SD2D3F_BJS_F |  | CTGRGAAAACCCGAAAGATCG |  | this study |  |
|  | 28SD2D3R_BJS_R |  | GTCCTGAAAGTACCCAAAGC |  | this study |  |
| CAD1 | CAD-Amel127F | CAD-hym-314F-B | GYGANACNCCNAGYCAYTG | 94°C/3:30 min; [94°C/30s; 59°C/ 30s; 72°C/90s]x3; [94°C/30s; 57.5°C/30s; 72°C/ 90s]x5; [94°C/30s; 54°C/30s; 72°C/90s]x8; 72°C/4 min | this study | Primer 127F works best with 252Ra or 252Rb (less degenerate). In some taxa 324R worked better with 127F, but alignments were trimmed to 252R. |
|  | CAD-Amel252Ra | CAD-hym-710R-A1 | GGTCCRTTRCTNARRAANAGNC |  | this study |  |
|  | CAD-Amel252Rb | CAD-hym-710R-A2 | GGTCCRTTRCTNARRAANAANC |  | this study |  |
|  | CAD-Amel324R | CAD-hym-926R | TGRTTYTGNGANGTCATRTARC |  | this study |  |
| CAD2 | CAD-Amel368F | Peas | CBGTDCARTTYCAYCCRGARCA | 94°C⁄ 2min; [94°C⁄30s; 61°C⁄15s; 72°C⁄45s]x30; 72°C⁄4min | this study | Primer pairs are 368F with 482R and 379F with 479R. Can be used as a nested PCR, see Figure 1 for placement of primers. |
|  | CAD-Amel482R | CarrotsA | ATVGGYAARAARTAHACYTTRT |  | this study |  |
|  | CAD-Amel379F | Broccoli | TTYCAYCCGGAGCAYAAYG |  | this study |  |
|  | CAD-Amel479R | Cabbage | TANACTTTGTCRGCCATYC |  | this study |  |
| AM2 | AM2-Amel244F | A-MAN-II-F1 | ATGCCNGAYGARTCNGTNTC | 94°C⁄ 2min; [94°C⁄ 30s; 61.5°C⁄ 20s; 72°C⁄ 45s]x3; [94°C/30s; 59.5°C/20s; 72°C/45s]x3; [94°C/30s; 57.5°C/20s; 72°C/45s]x8; [94°C/30s; 55°C/20s; 72°C/45s]x10; [94°C/30s; 52.5°C/20s; 72°C/45s]x12; 72°C⁄ 5 min | this study | Either forward can work with the reverse primer. Touchdown protocol helps specificity as well as the addition of 5% DMSO to the PCR reaction. |
|  | AM2-Amel356F | A-MAN-II-F2 | CCNAARGTNTGYTGYCARTT |  | this study |  |
|  | AM2-Amel484R | A-MAN-II-R | CARTARTGRTCRTCYCTRTC |  | this study |  |
| RPS23 | RPS23-34F | RPS23_21F | ACVMGVTGGAAGGCYAATCC | 94°C/ 5min; [94°C/ 30s; 61°C/ 30s; 72°C/ 60s]x3; [94°C/ 30s; 58°C/ 30s; 72°C/60s]x3; [94°C/ 30s; 54°C/ 30s; 72°C/ 60s]x8; 72°C⁄ 5 min | Lohse et al. (2011) | The forward primer can pair with either reverse primer. The touchdown helps specificity. Addition of 5% DMSO increases specificity. |
|  | RPS23-104R | RPS23_21R | ATGACCYTTACGHCCRAATCC |  | Lohse et al. (2011) |  |
|  | RPS23-107R | RPS23_21Rb | CCDACRGCRTGACCYTTACG |  | this study |  |
| 16S | 16SDAF |  | CACCTGTTTATCAAAAACAT | see Deans et al. (2006) | Dowton & Austin (1994) |  |
|  | 16SDAR |  | CTGCGATTTGAACTCAAATC |  | Dowton & Austin (1994) |  |
| COI | COI-LCO1490 |  | GGTCAACAAATCATAAAGATATTGG | see Namin et al. (2014)  see Schulmeister et al. (2002) | Folmer et al. (1994) |  |
|  | CO1-HCO2198 |  | TAAACTTCAGGGTGACCAAAAAATCA |  | Folmer et al. (1994) |  |
|  | COI lco hym |  | CAAATCATAAAGATATTGG |  | Schulmeister et al. (2002) |  |
|  | COI hco outout |  | GTAAATATATGRTGDGCTC |  | Schulmeister et al. (2002) |  |
