# Supplementary material for: Multi-gene phylogeny and divergence estimations for Evaniidae (Hymenoptera)
Source: PeerJ. 2019 Apr 4;7:e6689. doi: 10.7717/peerj.6689 (PMC6451838; doi:10.7717/peerj.6689)
Supplement: Table S3 — Highlighted genes indicate that the test for base composition homogeneity across taxa was falsified. Statistics are provided below the table. [file peerj-07-6689-s005.docx]

**Table S2**. **Average nucleotide composition (%) across genes**. Highlighted genes indicate that the test for base composition homogeneity across taxa was falsified. Statistics are provided below the table.

| **Genes:** | **T** | **C** | **A** | **G** |
| --- | --- | --- | --- | --- |
| 16S | 37.4 | 8.7 | 38.3 | 15.6 |
| 28S | 17.0 | 34.4 | 12.8 | 35.8 |
| AM2 | 22.8 | 29.1 | 22.7 | 25.4 |
| CAD1 | 24.3 | 21.5 | 31.1 | 23.2 |
| CAD2 | 25.2 | 22.5 | 29.1 | 23.2 |
| CO1 | 39.3 | 18.7 | 30.1 | 11.9 |
| RPS23 | 20.4 | 23.4 | 27.7 | 28.4 |

There was a significant difference in nucleotide composition across taxa for *AM2* (*χ*^2^= 368.819, *df*=120; *P* = 0.000000000) and *CO1* (*χ*^2^= 562.535, *df*=225; *P* = 0.00000000). There was no significant difference in nucleotide composition across taxa for *16S* (*χ*^2^ = 76.014, *df*=138; *P* = 0.99999599), *28S* (*χ*^2^ = 60.507, *df*=198; *P* = 1.00000000), *CAD1* (*χ*^2^ = 120.417, *df*=183; *P* = 0.99989499), *CAD2* (*χ*^2^ = 113.315, *df*=177; *P* = 0.99994532), and *RPS23* (*χ*^2^ = 105.386, *df*=183; *P* = 0.99999920).
