# Supplementary material for: Multi-gene phylogeny and divergence estimations for Evaniidae (Hymenoptera)
Source: PeerJ. 2019 Apr 4;7:e6689. doi: 10.7717/peerj.6689 (PMC6451838; doi:10.7717/peerj.6689)
Supplement: Table S4 — Monophyletic clades are marked with an x, which is shaded grey if the posterior probability was 0.95 or greater. N/A indicates that there was a missing taxon so the clade could not be recovered. A “C” preceding a taxon name in a cell indicates that a congruent clade was recovered even though the indicated taxon was missing. Taxon Abbreviations are as follows: Mic (Micrevania); Bra (Brachygaster); Pro (Prosevania); Tri (Trissevania); Eva (Evania); Zeu (Zeuxevania); Par (Parevania); CEv (Evaniscus); Dec (Decevania); Sem (Semaeomyia); Hyp (Hyptia); Alo (Alobevania); LEv (Evaniella); Sze (Szepligetella); Aca (Acanthinevania). [file peerj-07-6689-s006.docx]

**Table S3**. **Recovered clades across individual genes and concatenated analyses (All genes)**. Monophyletic clades are marked with the posterior probability, which is shaded grey if 0.95 or greater. N/A indicates that there was a missing taxon so the clade could not be recovered. A “C” preceding a taxon name in a cell indicates that a congruent clade was recovered even though the indicated taxon was missing. Taxon Abbreviations are as follows: Mic (*Micrevania*); Bra (*Brachygaster*); Pro (*Prosevania*); Tri (*Trissevania*); Eva (*Evania*); Zeu (*Zeuxevania*); Par (*Parevania*); CEv (*Evaniscus*); Dec (*Decevania*); Sem (*Semaeomyia*); Hyp (*Hyptia*); Alo (*Alobevania*); LEv (*Evaniella*); Sze (*Szepligetella*); Aca (*Acanthinevania*).

|  |  | **Genera recovered** | | | | | | | | | | | | | | | | | | | | | | | |
| --- | --- | --- | --- | --- | --- | --- | --- | --- | --- | --- | --- | --- | --- | --- | --- | --- | --- | --- | --- | --- | --- | --- | --- | --- | --- |
| **Assoc. Figure** | **Gene Region** | **Mic** | **Bra** | | **Pro** | | **Tri** | | **Eva** | **Zeu** | | **Par** | | **CEv** | **Dec** | | **Sem** | | **Hyp** | | **Alo** | **LEv** | | **Sze** | **Aca** |
| **S1** | **28S** |  | **1.0** | | **1.0** | | **N/A** | | **0.94** |  | |  | |  | **1.0** | | **1.0** | | **1.0** | | **N/A** | **1.0** | |  |  |
| **S2** | **16S** | **0.96** | **0.98** | | **1.0** | | **N/A** | |  |  | |  | | **0.96** | **1.0** | |  | | **0.75** | | **N/A** | **1.0** | |  | **x** |
| **S3** | **COI** | **1.0** | **1.0** | | **1.0** | | **N/A** | | **0.61** | **0.79** | |  | |  | **1.0** | | **1.0** | | **1.0** | | **N/A** | **1.0** | |  | **x** |
| **S4** | **CAD1** | **N/A** | **1.0** | | **1.0** | | **N/A** | | **1.0** |  | |  | | **N/A** | **1.0** | | **0.99** | | **1.0** | | **N/A** | **1.0** | |  |  |
| **S5** | **CAD2** |  | **1.0** | | **1.0** | | **1.0** | | **1.0** |  | |  | | **N/A** | **1.0** | | **1.0** | | **1.0** | | **N/A** |  | |  |  |
| **S6** | **RPS23** | **N/A** | **0.95** | | **N/A** | | **1.0** | |  |  | |  | | **0.81** | **0.98** | | **0.76** | | **0.81** | | **N/A** |  | |  |  |
| **S7** | **AM2** | **N/A** | **N/A** | | **0.99** | | **N/A** | | **N/A** | **N/A** | | **0.94** | | **N/A** | **1.0** | | **N/A** | | **0.99** | | **N/A** |  | |  |  |
| **2** | **All** |  | **1.0** | | **1.0** | | **1.0** | | **1.0** |  | |  | | **1.0** | **1.0** | | **1.0** | | **1.0** | | **N/A** | **1.0** | |  |  |
|  |  | **Higher Level Clades Recovered** | | | | | | | | | | | | | | | | | | | | | | | |
| **Assoc. Figure** | **Gene Region** | **Sze + Aca** | | **LEv + Sze + Aca** | | **Alo + Eva + Sze + Aca** | | **CEv + Dec + Rot + Sem + Hyp** | | | **Zeu + Par + Pap** | | **Bra + Pro** | | | **All but Bra/ Pro/ Mic** | | **Tri + Eva** | | **Tri + Evan**  **Zeu + Par + Pap** | | |  |  |  |
| **S1** | **28S** | **0.94** | | **1.0** | | **0.88** | |  | | | **1.0** | |  | | |  | |  | |  | | |  |  |  |
| **S2** | **16S** |  | |  | |  | |  | | |  | |  | | |  | |  | |  | | |  |  |  |
| **S3** | **COI** | **1.0** | | **0.99** | | **N/A** | |  | | | **1.0** | |  | | |  | | **0.82** | |  | | |  |  |  |
| **S4** | **CAD1** | **1.0** | |  | |  | | **0.94 C (CEv)** | | | **1.0** | |  | | |  | | **N/A** | | **N/A** | | |  |  |  |
| **S5** | **CAD2** | **1.0** | | **0.91** | |  | | **0.66 C (CEv)** | | | **1.0** | | **0.99** | | | **0.57** | |  | |  | | |  |  |  |
| **S6** | **RPS23** | **0.71** | |  | | **N/A** | |  | | | **0.96** | |  | | |  | |  | |  | | |  |  |  |
| **S7** | **AM2** | **0.75** | |  | | **0.72** | |  | | | **N/A** | | **N/A** | | |  | |  | |  | | |  |  |  |
| **2** | **All** | **1.0** | | **1.0** | | **1.0** | | **1.0** | | | **1.0** | | **1.0** | | | **1.0** | | **1.0** | | **0.98** | | |  |  |  |
