# Supplementary material for: Multi-gene phylogeny and divergence estimations for Evaniidae (Hymenoptera)
Source: PeerJ. 2019 Apr 4;7:e6689. doi: 10.7717/peerj.6689 (PMC6451838; doi:10.7717/peerj.6689)
Supplement: Figures S1–S7 — Bayesian analysis of each gene individually for Evaniidae. Posterior probabilities are listed beside each node. [file peerj-07-6689-s007.docx]

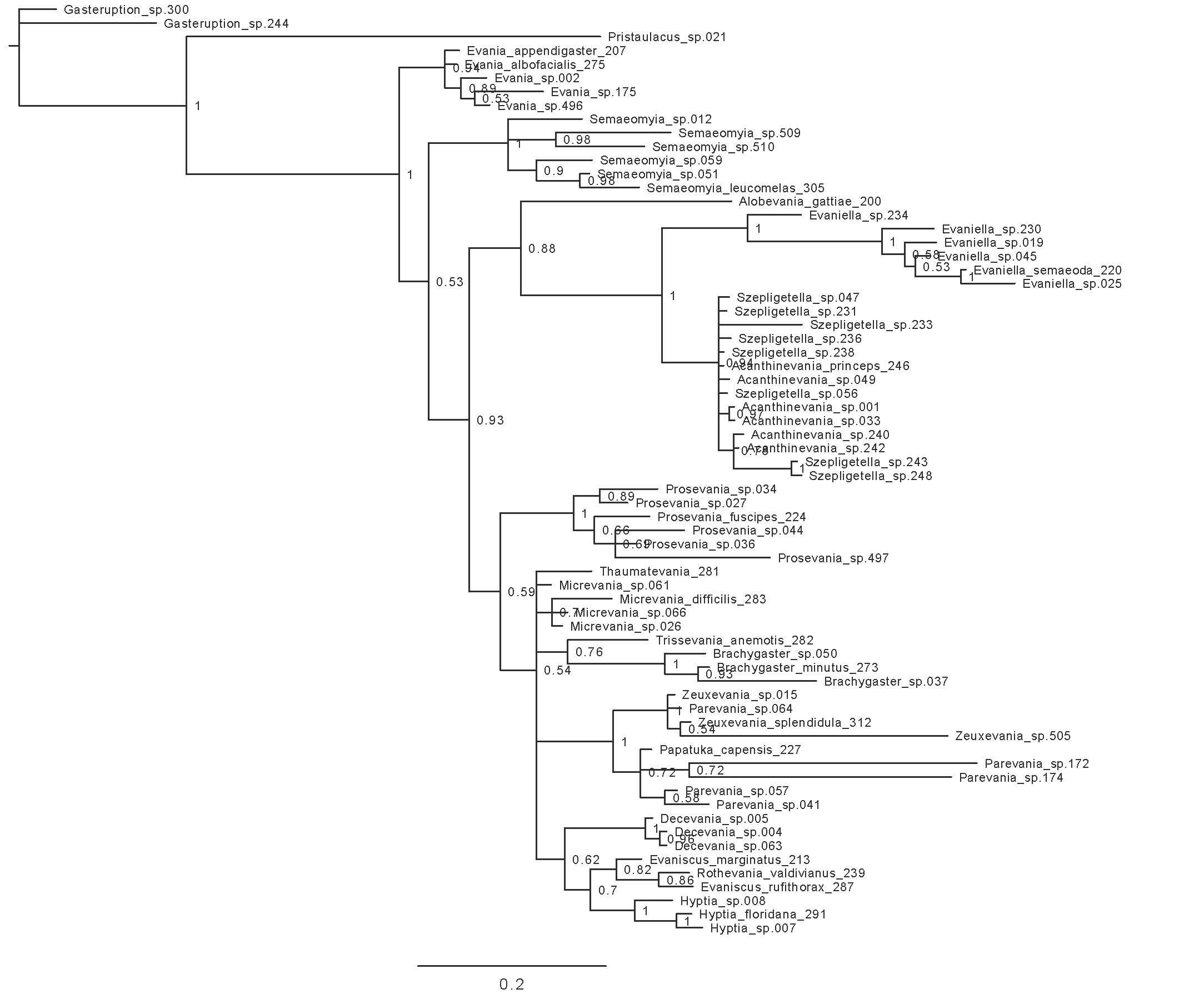


**Figure S1**. Bayesian analysis of 28S rDNA gene for Evaniidae. Posterior probabilities are listed beside the node.


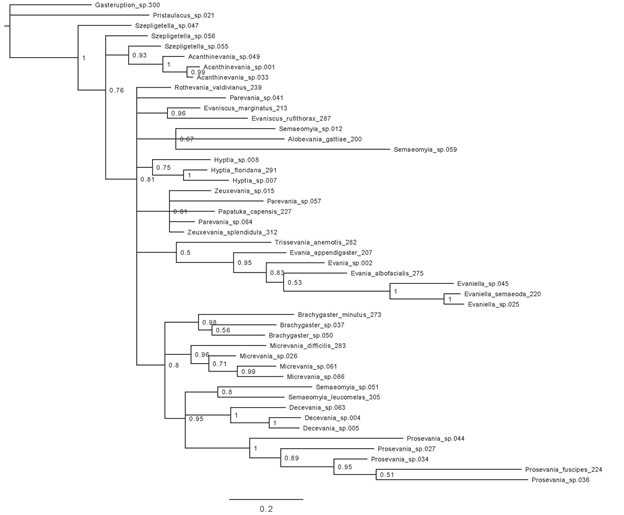
**Figure S2**. Bayesian analysis of 16S rDNA gene for Evaniidae. Posterior probabilities are listed beside the node.


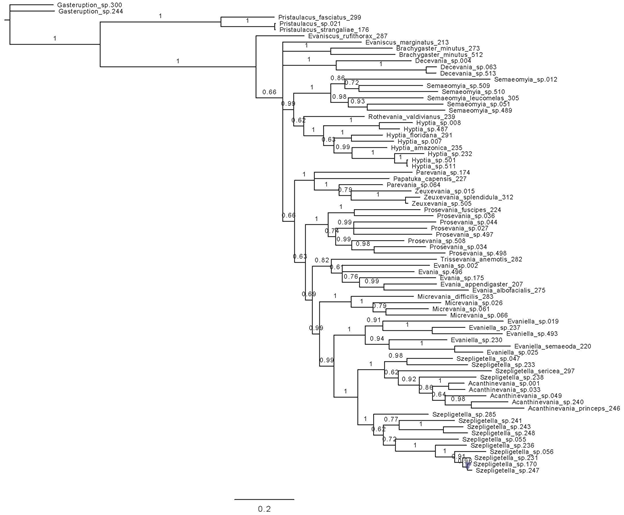
**Figure S3**. Bayesian analysis of COI gene for Evaniidae. Posterior probabilities are listed beside the node.


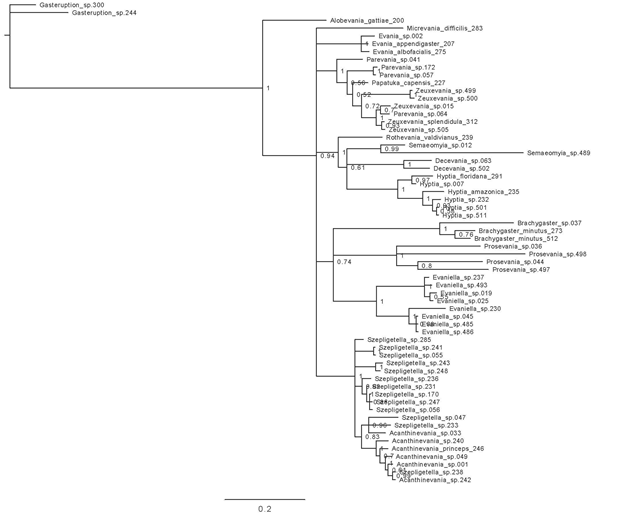
**Figure S4**. Bayesian analysis of CAD1 gene for Evaniidae. Posterior probabilities are listed beside the node.


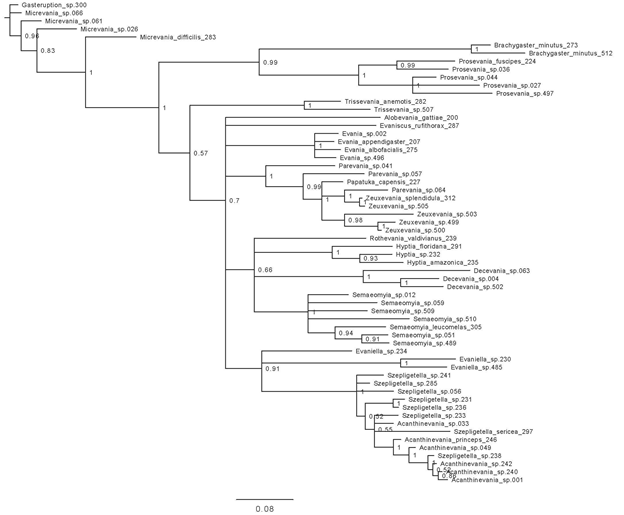
**Figure S5**. Bayesian analysis of CAD2 gene for Evaniidae. Posterior probabilities are listed beside the node.


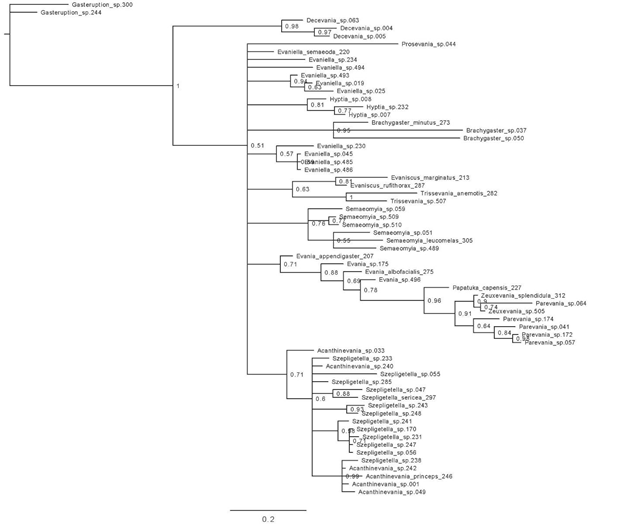
**Figure S6**. Bayesian analysis of RPS23 gene for Evaniidae. Posterior probabilities are listed beside the node.


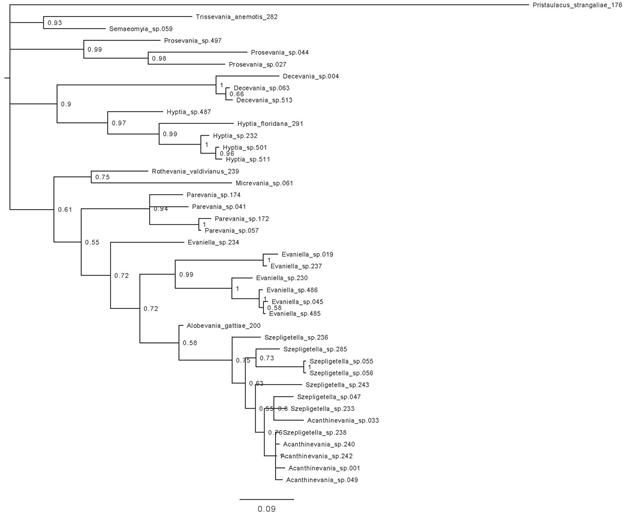
**Figure S7**. Bayesian analysis of AM2 gene for Evaniidae. Posterior probabilities are listed beside the node.
