# Supplementary material for: Multi-gene phylogeny and divergence estimations for Evaniidae (Hymenoptera)
Source: PeerJ. 2019 Apr 4;7:e6689. doi: 10.7717/peerj.6689 (PMC6451838; doi:10.7717/peerj.6689)
Supplement: Figures S8–S13 — Figures that illustrate the unique sequence motifs (RPS23 and 28S rDNA) for certain lineages of Evaniidae. [file peerj-07-6689-s008.docx]

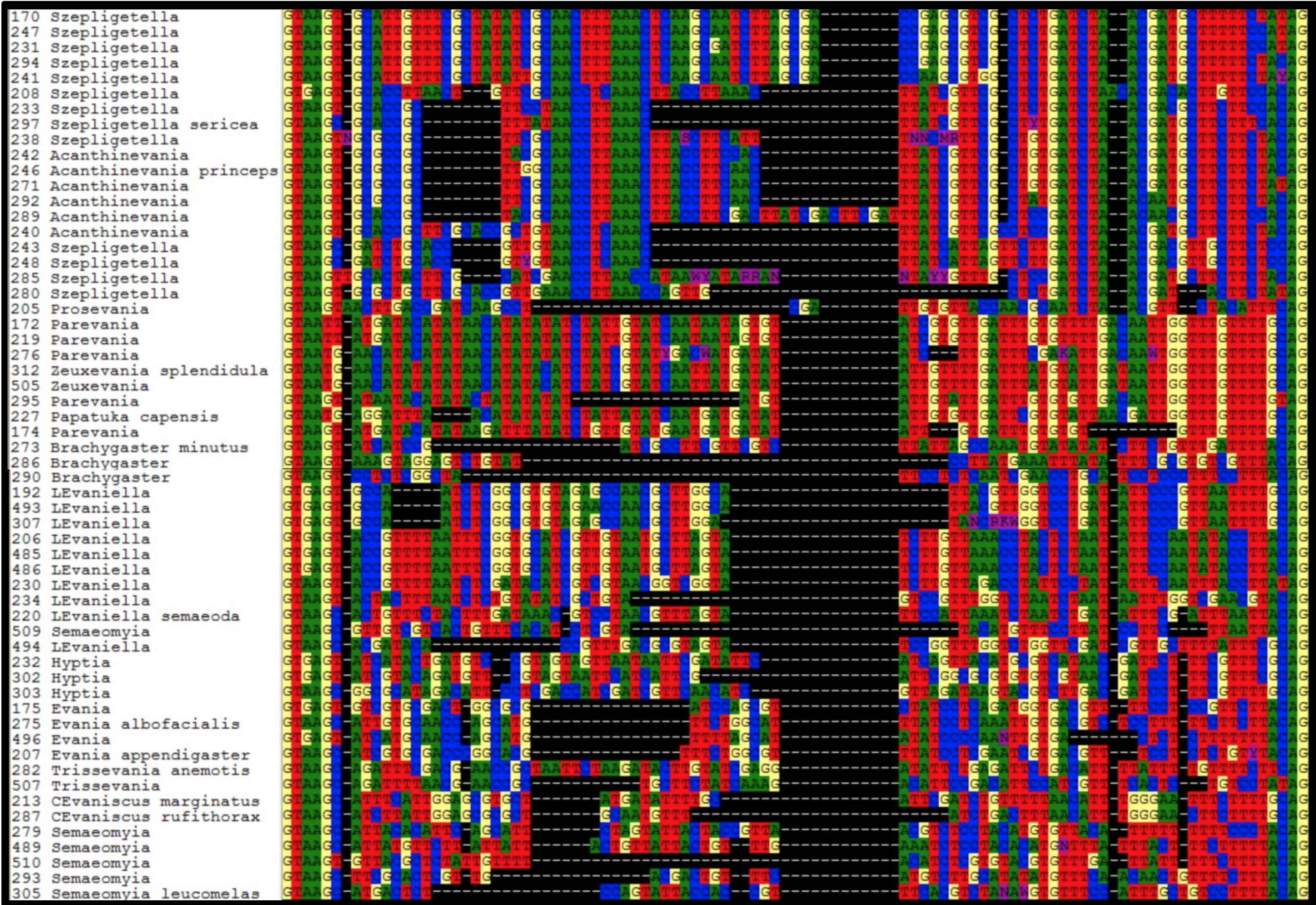


**Figure S8**. RPS23 intron aligned for all taxa within Evaniidae. Species of *Decevania* were removed due to high sequence length variation to facilitate better visualization of sequence motifs across all taxa.


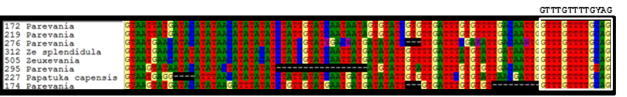


**Figure S9**. RPS23 intron aligned for *Zeuxevania*, *Parevania*, and *Papatuka*. Box showing sequence motif.


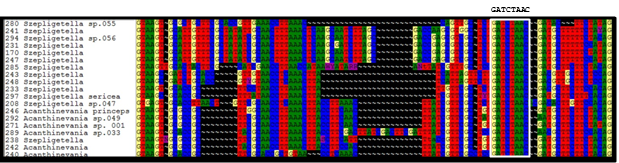
**Figure S10**. RPS23 aligned intron for *Acanthinevania* and *Szepligetella*. Box showing sequence motif.


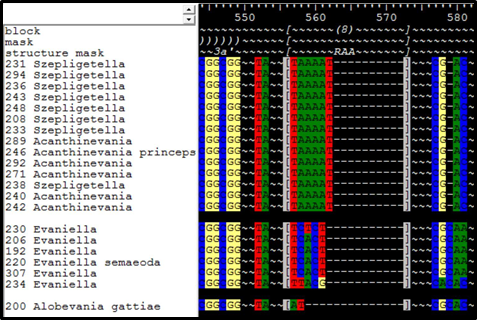


**Figure S11**. Part of 28S alignment for *Acanthinevania*, *Szepligetella*, *Evaniella*, and *Alobevania*. Note the diagnostic TAAAAT motif in RAA8 for *Acanthinevania* and *Szepligetella*.


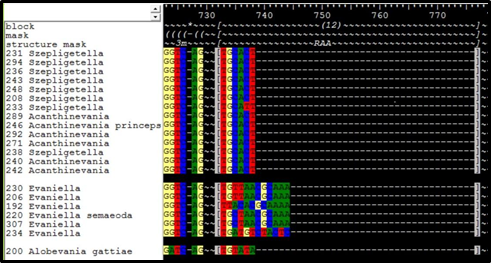


**Figure S12**. Part of 28S alignment for *Acanthinevania*, *Szepligetella*, *Evaniella*, and *Alobevania*. Note the diagnostic TGCAYT motif in RAA12 for *Acanthinevania* and *Szepligetella*.


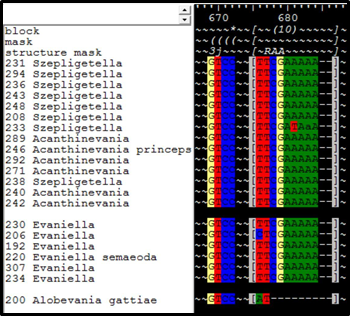


**Figure S13**. Part of 28S alignment for *Acanthinevania*, *Szepligetella*, *Evaniella*, and *Alobevania*. Note the diagnostic YTCGAWAAA motif in RAA10 for *Acanthinevania*, *Szepligetella*, and *Evaniella*.
