# Supplementary material for: Multi-gene phylogeny and divergence estimations for Evaniidae (Hymenoptera)
Source: PeerJ. 2019 Apr 4;7:e6689. doi: 10.7717/peerj.6689 (PMC6451838; doi:10.7717/peerj.6689)
Supplement: Figure S14 — Posterior probabilities are listed beside the relevant node. The 95% HDP for all clades is listed in Table 2. The scale is in millions of years. [file peerj-07-6689-s009.docx]

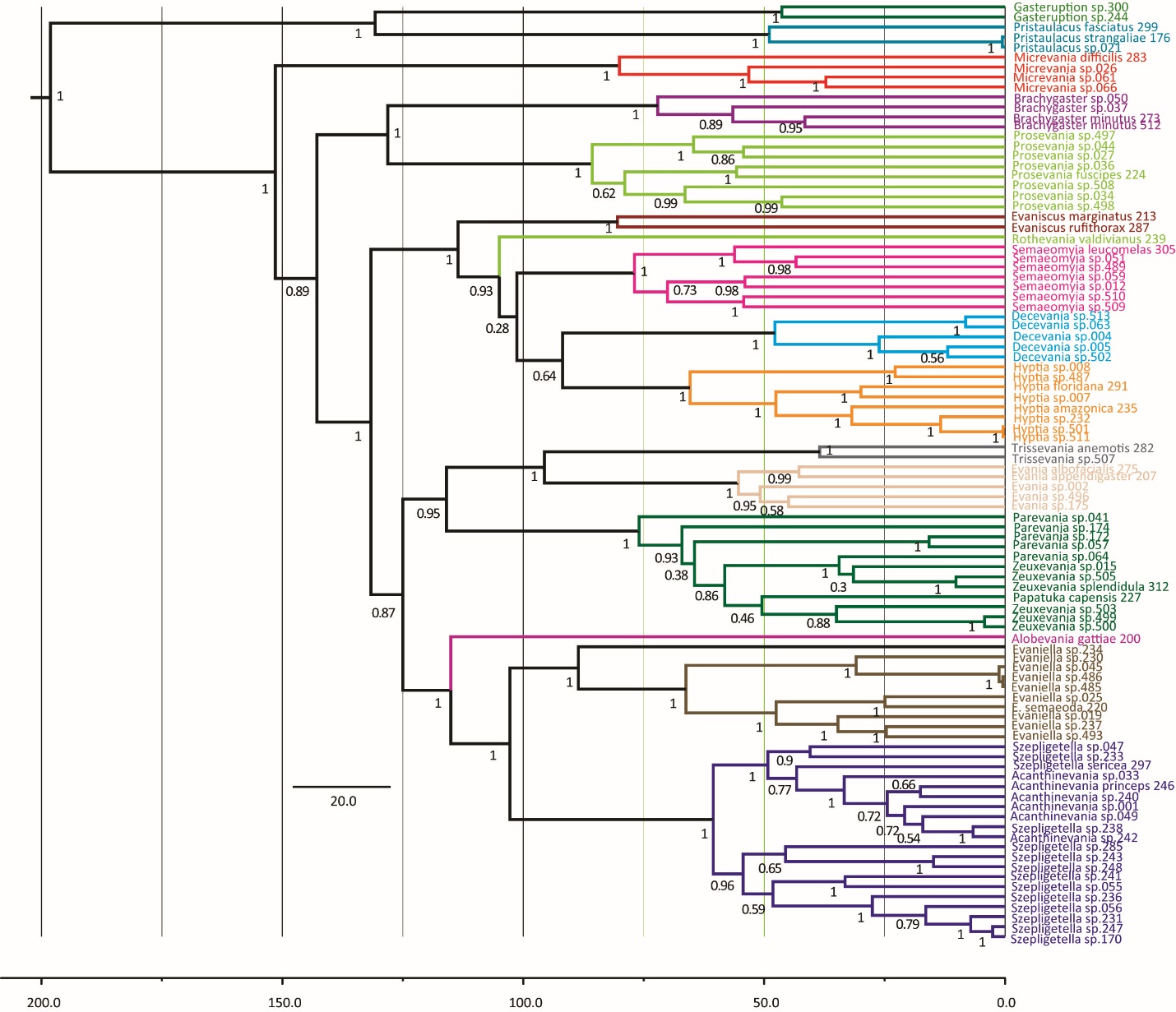


**Figure S14**. Estimated divergence times for Evaniidae under a normal distribution. Posterior probabilities are listed beside the relevant node. The 95% HDP for all clades is listed in Table 2. The scale is in millions of years.
