# Supplementary material for: Multi-gene phylogeny and divergence estimations for Evaniidae (Hymenoptera)
Source: PeerJ. 2019 Apr 4;7:e6689. doi: 10.7717/peerj.6689 (PMC6451838; doi:10.7717/peerj.6689)
Supplement: Figure S15 — Maximum Likelihood analyses of the concatenated dataset. Bootstrap values are listed beside each node. [file peerj-07-6689-s010.docx]

**
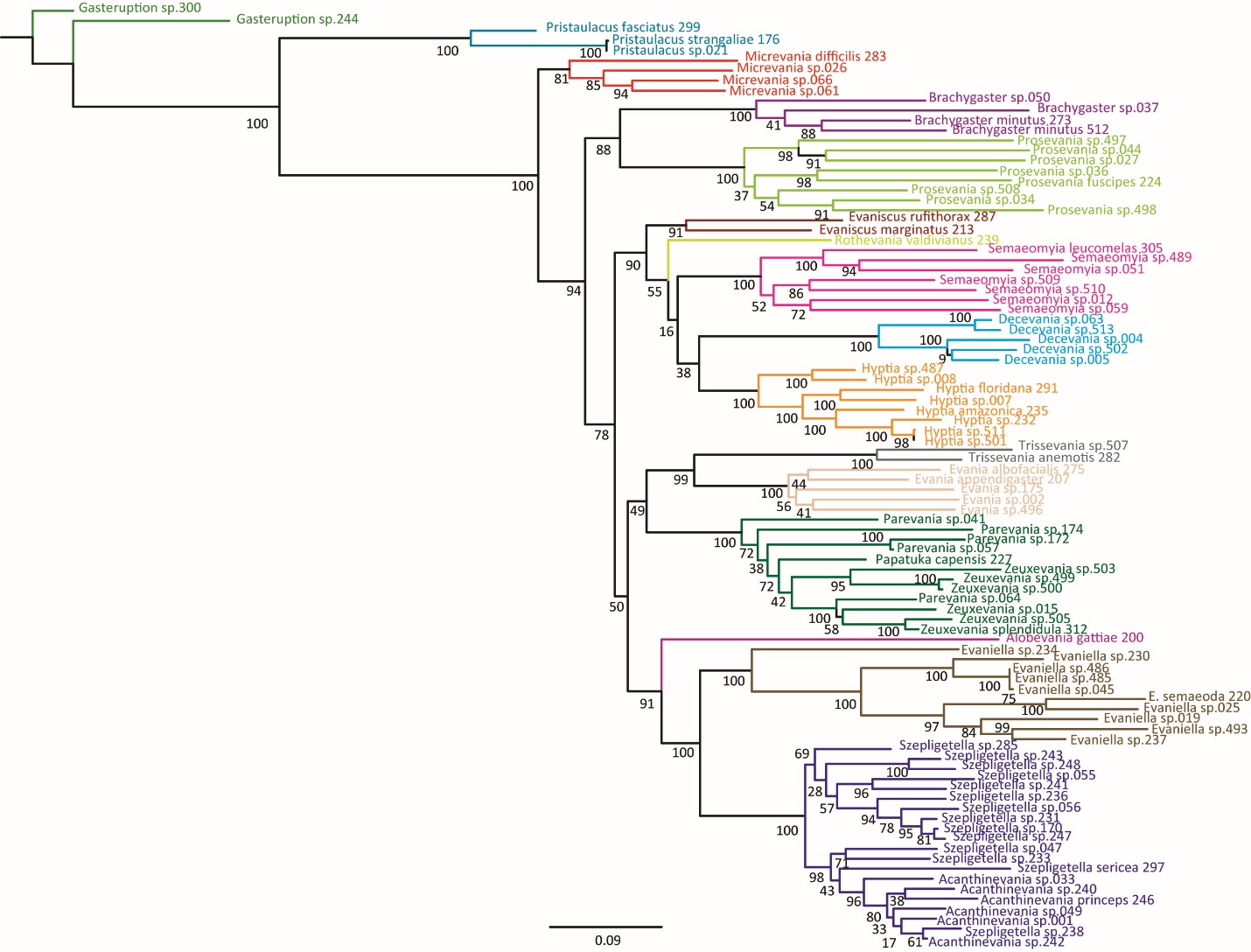
Figure S15**. Maximum Likelihood analyses of the concatenated dataset. Bootstrap Values are listed beside each node.
