# Supplementary material for: Multi-gene phylogeny and divergence estimations for Evaniidae (Hymenoptera)
Source: PeerJ. 2019 Apr 4;7:e6689. doi: 10.7717/peerj.6689 (PMC6451838; doi:10.7717/peerj.6689)
Supplement: Supplemental Information 2 — Fossil calibration points and references for the divergence time estimation analyses. [file peerj-07-6689-s011.docx]

**Fossil Calibrations**

All dates for geological time periods are based on the published dates by the International Commission on Stratigraphy (Cohen et al., 2013). Each fossil was assigned to the crown group for which they belonged based on shared synapomorphies as noted below. Analysis 1 was performed under a lognormal distribution and Analysis 2 was performed using a normal distribution for calibration priors, as justified below.

**Calibration 1** – Evaniidae

To calibrate our ingroup, we chose to use the oldest known fossil confidently placed within Evaniidae (Peñalver et al., 2010), *Cretevania concordia* Rasnitsyn, Jarzembowski, & Ross, 1998, known from the Upper Hauterivian of the Cretaceous, beginning at 132.9 mya (Rasnitsyn et al., 1998). Placement within the Evaniidae is based on the following synapomorphies: dorsal insertion of the metasoma; tubular petiole; 11 flagellomeres; and large head adpressed to prothorax (Peñalver et al., 2010; Deans and Huben, 2003). For analysis 1, under a lognormal prior distribution, we set the mean at 1.2, Stdev at 1, and used an offset of 134 (95% HPD: 134.5-157.6). For analysis 2, the maximum bound was set as the mid Jurassic, for which there are no known fossils within Evaniidae, but there are fossils within Evanioidea (for example, Preaulacidae). We chose the start of the Bethonian of the Middle Jurassic (166.1 mya) for the maximum bound, as it includes the period with the oldest known Evanioid fossils. Thus, the mean was set to 149.5, with a standard deviation set to 8.4 to achieve a 95% HPD of 133.0 – 166.0.

**Calibration 2** – *Evaniella*

*Evaniella eocenica* Sawoniewicz & Kupryjanowicz from the mid Eocene Baltic amber is the only known fossil in this genus (Sawoniewicz and Kupryjanowicz, 2003). Placement within *Evaniella* is based on the following synapomorphies: Loss of cross veins in the apical area of the fore wing (i.e., with at 7 closed cells proximally); linear 1RS, intersecting with SC+R near pterostigma (Deans and Huben 2003). And these diagnostic characters: hind coxa close to mid coxa (closer than twice the length of the mid coxa), and face without longitudinal striae (Deans and Huben, 2003; Sawoniewicz and Kupryjanowicz, 2003). According to Ritzkowski (1997), Baltic amber is dated to 44.1 ± 1.1 mya, thus placing the fossil in the Lutetian of the Eocene. Using this as the lower bound, we set the mean at 1.61, Stdev at 0.8, and used an offset of 45 to achieve a 95% HPD of 46.0 – 69.0 for analysis 1. For analysis 2, we set a maximum bound at 132.9 mya, which is the lower bound set for Evaniidae from calibration 1. With bounds at 44.1 – 132.9 mya, we set the mean at 88.5 and Stdev at 22.7 to achieve a 95% HPD of 44.0 – 133.0 for analysis 2.

**Calibration 3** – *Hyptia*

*Hyptia hennigi* Jennings, Krogmann & Priya was described from Baltic amber (Jennings et al., 2013), and thus has a minimum age of 44.1 mya as discussed for calibration 2. Placement within *Hyptia* is based on the following synapomorphy: loss of nearly all longitudinal veins and crossveins on fore wing (i.e., fore wing with only one cell complete, enclosed by tubular veins); and this diagnostic character: 11 flagellomeres (distinguishing it from *Decevania*) (Deans and Huben, 2003; Jennings et al., 2013). We set the mean at 1.61, Stdev at 0.8, and used an offset of 45 to achieve a 95% HPD of 46.0 – 69.0 for analysis 1*.* For analysis 2, we set a maximum bound at 132.9 mya, which is the lower bound set for Evaniidae from calibration 1, similar as was done for calibration 2. Thus, with bounds at 44.1 – 132.9 mya, we set the mean at 88.5 and Stdev at 22.7 to achieve a 95% HPD of 44.0 – 133.0 for analysis 2. Note that if this fossil was a stem fossil, we still believe it is better suited to constrain the MRCA of the sampled *Hyptia*, rather than the MRCA of *Hyptia* + *Decevania* given the uncertainty in this relationship, the long branch from that MRCA to the *Hyptia* clade, and our use of two different distributions that allow for uncertainty in the estimate. Further, using this fossil as a constraint for the MRCA of Hyptia + *Decevania* did not significantly change the estimate.

**Calibration 4** – *Pristaulacus* (Aulacidae)

*Pristaulacus velteni* Jennings and Krogmann was collected from Baltic amber (Jennings and Krogmann, 2009), and thus has a minimum age of 44.1 mya as discussed for calibration 2. Placement within *Pristaulacus* is based on the following synapomorphies: hind claws pectinate and occipital carina present (Jennings and Krogmann, 2009). We set the mean at 1.61, Stdev at 0.8, and used an offset of 45 to achieve a 95% HPD of 46.0 – 69.0 for analysis 1*.* For an upper bound, we chose the end of the Cretaceous (~145 mya) as there are no known aulacid fossils known from the Jurassic. This bound seems reasonable as there are extinct members of Aulacidae (*Hyptiogastrites electrinus* Cockerell), placed as sister to extant Aulacidae (Jennings et al., 2004), known from the Upper Albian of the Lower Cretaceous. For analysis 2, we set the mean at 94.6 and Stdev at 25.8 for a 95%HDP of 44.0 – 145.2.

**Calibration 5** – *Parevania*

*Parevania brevis*(Brues) was described from Baltic amber (Brues, 1933). Placement within *Parevania* is based on the following synapomorphies: forewing with seven complete cells, and forweing vein 1RS meeting Sc +R basal to the pterostigma. The calibration for *Parevania* is the same as calibration 2 for *Evaniella* for both analyses, as the maximum bound was also set as the lower bound for Evaniidae. Thus, we set the mean at 1.61, Stdev at 0.8, and used an offset of 45 to achieve a 95% HPD of 46.0 – 69.0 for analysis 1 and the mean at 88.5 and Stdev at 22.7 to achieve a 95% HPD of 44.0 – 133.0 for analysis 2.

**Calibration 6** – *Semaeomyia*

*Semaeomyia dominicana* [(Nel, Martinez-Delclos & Azar)](http://evanioidea.info/public/ref/show/30343) was described from Dominican amber, although the exact age of the fossil is unknown (Nel et al., 2002). Placement within *Parevania* is based on the following synapomorphies: forewing with 3 closed cells, with first marginal cell absent; tarsal claws with the subapical tooth larger and stouter than the apical tooth (Deans and Huben, 2003). Dominican amber is late Early Miocene through early Middle Miocene (15-20 mya) (Iturralde-Vinent and MacPhee, 1999). Similar to other calibrations we set the upper bound as the lower for Evaniidae at 132.9 mya. Thus, we set the mean at 1.61, Stdev at 0.8, and used an offset of 21 to achieve a 95% HPD of 22.0 – 45.0 for analysis 1 and the mean at 76.5 and Stdev a 28.8 for a 95%HDP of 20.0 – 132.9.

**References for Calibrations**

Brues, C.T., 1933. The parasitic Hymenoptera of the Baltic amber. Part I. . Bernstein-Forschungen (Amber Studies), 3: 4-178.

Cohen, K.M., Finney, S.C., Gibbard, P.L. and Fan, J.X., 2013. The ICS international chronostratigraphic chart. Episodes, 36(3): 199-204.

Iturralde-Vinent, M. and MacPhee, R.D.E., 1999. Paleogeography of the Caribbean region: Implications for Cenozoic biogeography, Bulletin of the AMNH. No. 238, pp. 95.

Jennings, J.T., Austin, A.D. and Stevens, N.B., 2004. *Hyptiogastrites electrinus* Cockerell, 1917, from Myanmar (burmese) amber: Redescription and its placement within the Evanioidea (Insecta: Hymenoptera). Journal of Systematic Palaeontology, 2(02): 127-132.

Jennings, J.T. and Krogmann, L., 2009. A new species of *Pristaulacus* Kieffer (Hymenoptera: Aulacidae) from Baltic amber. Insect Syst. Evol., 40(2): 201-207.

Jennings, J.T., Krogmann, L. and Priya, P., 2013. Happy birthday Willi Hennig!-*Hyptia hennigi* sp. nov.(Hymenoptera: Evaniidae), a fossil ensign wasp from Eocene Baltic amber. Zootaxa, 3731: 395-398.

Nel, A., Martínez-Delclòs, X., and Azar, D. 2002. A new ensign-fly from the Lower-Middle Miocene Dominican amber (Hymenoptera, Evaniidae). Bulletin de la Societe Entomologique de France, 107(3), 217-221.

Peñalver, E., Ortega-Blanco, J., Nel, A. and Delclòs, X., 2010. Mesozoic Evaniidae (Insecta: Hymenoptera) in Spanish amber: Reanalysis of the phylogeny of the Evanioidea. Acta Geologica Sinica, 84(4): 809-827.

Rasnitsyn, A.P., Jarzembowski, E.A. and Ross, A.J., 1998. Wasps (Insecta: Vespida=Hymenoptera) from the Purbeck and Wealden (Lower Cretaceous) of southern England and their biostratigraphical and palaeoenvironmental significance. Cretaceous Res., 19(3–4): 329-391.

Ritzkowski, S., 1997. K-Ar-Altersbestimmungen der bernsteinführenden sedimente des samlandes (Paläogen, Bezirk Kaliningrad). Metalla (Bochum), 66: 19-23.

Sawoniewicz, J. and Kupryjanowicz, J., 2003. *Evaniella eocenica* sp. nov. from the Baltic amber (Hymenoptera: Evaniidae). Acta Zool. Cracov., 46 (Suppl. - Fossil Insects): 267-270.
